# A Large Cavernous Sinus Giant Cell Tumor Invading Clivus and Sphenoid Sinus Masquerading as Meningioma: A Case Report and Literature Review

**DOI:** 10.3389/fsurg.2022.861739

**Published:** 2022-03-24

**Authors:** Shasha Hu, Shaowen Cheng, Yu Wu, Yanyan Wang, XinNian Li, Jiaxuan Zheng, Jiao Li, Lei Peng, Jian Yang

**Affiliations:** ^1^Department of Pathology, Hainan General Hospital, Hainan Affiliated Hospital of Hainan Medical University, Haikou, China; ^2^Trauma Center, The First Affiliated Hospital of Hainan Medical University, Haikou, China; ^3^Department of Wound Repair, First Affiliated Hospital of Hainan Medical University, Haikou, China; ^4^Key Laboratory of Emergency and Trauma Ministry of Education, Hainan Medical University, Haikou, China

**Keywords:** case report, giant cell tumors, cavernous sinus, clivus, sphenoid sinus

## Abstract

Giant cell tumor (GCT) of the bone is a rare benign, locally aggressive tumor that occurs in the epiphysis of long bones, especially the lower femur and the upper tibia. GCT of the bone of cranial origin is very rare, accounting for 1% of all GCT of the bone. We report the diagnosis, treatment, and immunohistochemistry of a rare case of intracranial GCT of the bone. We also review and summarize the imaging features, diagnostic markers, and current major treatment options for GCT of the bone. Our case and literature review emphasizes the importance of considering the full picture when making a diagnosis, rather than relying on imaging alone to make the diagnosis.

## Introduction

Giant cell tumors (GCTs) of the bone are benign osteolytic tumors with locally aggressive and rare tendency to metastasize, and more than 70% of cases tend to occur in young patients aged 20–40 years and have a female predominance (1–3). GCTs are thought to originate from neoplastic non-osteogenic stromal cells of the bone marrow and are characterized histologically by numerous multinucleated giant cells (MNGC) that are diffusely distributed among a background of mononuclear histiocytic cells (MNHC) and giant cell tumor stroma cells (GCTSC) (4). These tumors account for ~3–5% of all primary bone tumors (5, 6) and usually arise from the metaphysis of a long bone, or at an apophysis, especially at the distal femur, proximal tibia, or distal radius (7).GCTs have a tendency to involve the skull, most frequently the sphenoid bone, followed by the temporal bone, and account for <1% of all bone GCTs (8, 9). The preference for the sphenoid and temporal bone is due to the endochondral ossification histogenesis compared with the intramembranous ossification of the other cranial bones (10, 11). Here, we reported a rare case of the clivus GCT invading the clivus, cavernous sinus, and sphenoid sinus, and was misdiagnosed as meningioma.

## Case Presentation

**Figure 1:**
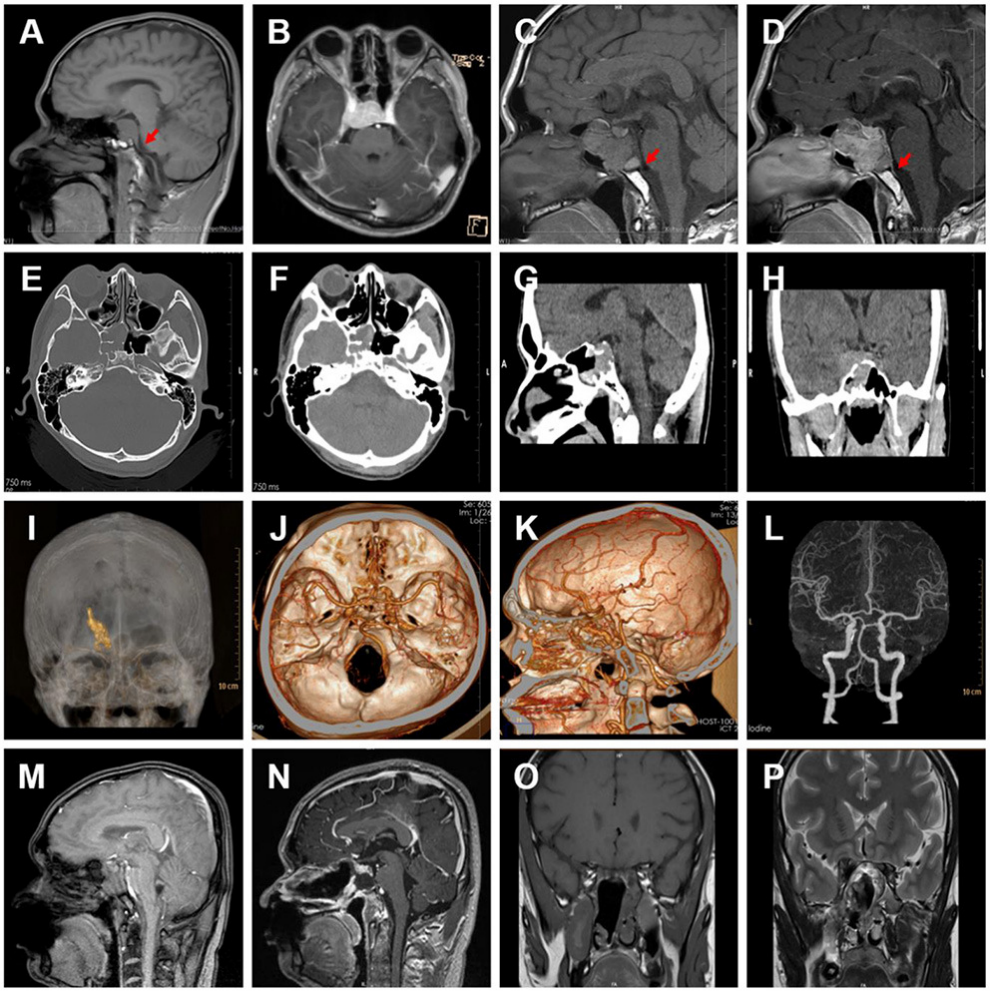
**(A)** MRI T1-weighted image from 2018-08-01, with the meningeal caudal sign in the red arrow. **(B)** MRI T1-weighted image from 2018-08-13. **(C)** MRI T1-weighted image from 2021-06-01. **(D)** MRI T1-weighted enhanced image from 2021-06-01, with the meningeal caudal sign in the red arrow. **(E–H)** CT images with the bone window. **(I–K)** 3D reconstruction of CT scan shows the tumor was posterior to the basilar artery, anterior to the hypophysial stalk, and surrounded by the internal carotid artery. **(L)** Pre-operative CTA contrast imaging. **(M–P)** Postoperative images.

In August 2018, a 20-year-old male was admitted to our hospital because of limited movement in his right eye for 2 weeks. His physical examination showed a good general state of health, but the right eye was limited in abduction (damage to the right abductor nerve). The patient was evaluated with a computerized tomogram (CT) and magnetic resonance imaging (MRI) of the brain. CT revealed a mass of slightly high density in the saddle area and cavernous sinus area that was irregular in size, about 2.4 × 2.1 × 1.8 cm, with osteolytic and distending changes, localized bone resorption in the saddle and pterygoid sinus wall, and close relationship between the lesion and the right dura mater (Figure 1). Enhancement MRI scans showed a heterogeneous enhancement of the lesion and dural tail sign without peritumoral edema in the right cavernous sinus. The characteristic meningeal tail sign and meningeal sign are shown in the red circle (Figure 1). The right lateral internal carotid artery (ICA) was identified within the lesion. The tumor had isointensity on the T1-weighted MRI scan and T2-weighted MRI scan. The tumor also had isointensity on diffusion-weighted imaging (DWI) scan and T2-weighted MRI scan. The diagnosis of the right cavernous sinus meningioma was considered in the context of history, imaging evidence, and physical examination, but the patient refused surgical treatment. During the subsequent 2-year follow-up, the patient was treated with gamma-knife and did not develop meningeal irritation during treatment.

After 2 years, in May 2021, the patient was admitted by the emergency department with a sudden onset of dizziness with left upper body weakness for 2 days. Ophthalmologic examination showed that the pupils were unequal in size bilaterally, the left pupil was 3 mm in diameter with a sensitive reflex to light and the right pupil was 5 mm with a loss of reflex to light (damage to the right articular nerve). The remaining neurological examination was normal. CT revealed an osteolytic, distending lesion, an irregular tumor that was 4.6 × 3.5 × 3.0 cm in size, involving the saddle area and cavernous sinus area, and a right thalamic hemorrhage, 2.0 × 0.9 cm in size in the cross-section (Figure 1). We constructed a 3D reconstruction of this tumor in preparation for subsequent surgical treatment (Figure 1).

An endoscopic endonasal approach (EEA) was chosen as the surgical approach. Under neuroendoscopy, the nasal septum mucosal flap was made from the nasal septum, the mucosa of the nasal septum was separated, part of the bone of the nasal septum was bitten off, the bone of the upper slope area below the saddle base and the anterior lower wall of the pterygoid sinus was ground off, and part of the mucosa of the pterygoid sinus was removed, and the tumor was visible, with a tough texture and rich blood supply. The tumor was removed with a tumor clamp. Some of the tumors were removed with tumor clamp for pathology, some of the tumors were removed with suction, and the tumor was scraped from the base of the saddle and the left side of the right side of the pterygoid sinus and above the right side of the pterygoid sinus until no tumor was scraped out of the pterygoid sinus. However, the tumor was difficult to be resected because of the bleeding in the cavernous sinus. The size of the tumor was about 3.2 × 3.6 × 3.0 cm. It was solid, grayish-yellow in color with a clear border, having a general blood supply and a tough texture. The base of the saddle was found, and the dura of the saddle base and the upper slope was revealed. Gelatin sponge compression was applied to completely stop the bleeding, the saddle base was closed with bioprotein gel, the butterfly sinus was filled with gelatin sponge, the mucosal flap was repositioned, the blood in the nasal cavity was aspirated, and the right nasal cavity was filled with one strip of iodoform gauze to stop the bleeding. The operation was completed.

After surgery, the patient was conscious of intermittent headache and dizziness, but without nausea or vomiting. The answers were tangential, and the muscle strength and tone of the limbs were normal. The pupils were unequal in size bilaterally, the left pupil was 3 mm in diameter with a sensitive reflex to light and the right pupil was 5 mm with a loss of reflex to light. The patient had conjunctival congestion in the right eye (subsequent improvement), normal muscle tone in all four limbs, and limb muscle strength grade 5. Bilateral Babinski's sign was negative. The postoperative body temperature decreased from a maximum of 38–35.7°C during the follow-up treatment. At discharge, the patient had no fever, no cerebrospinal fluid nasal leakage, no cough or sputum, and smooth breathing. Diet, sleep, defecation, urination, and blood pressure were normal. The patient had no headache, was lucid, answered tangential questions, spoke fluently, and had grade 5 limb muscle strength and normal visual field vision.

**Figure 2:**
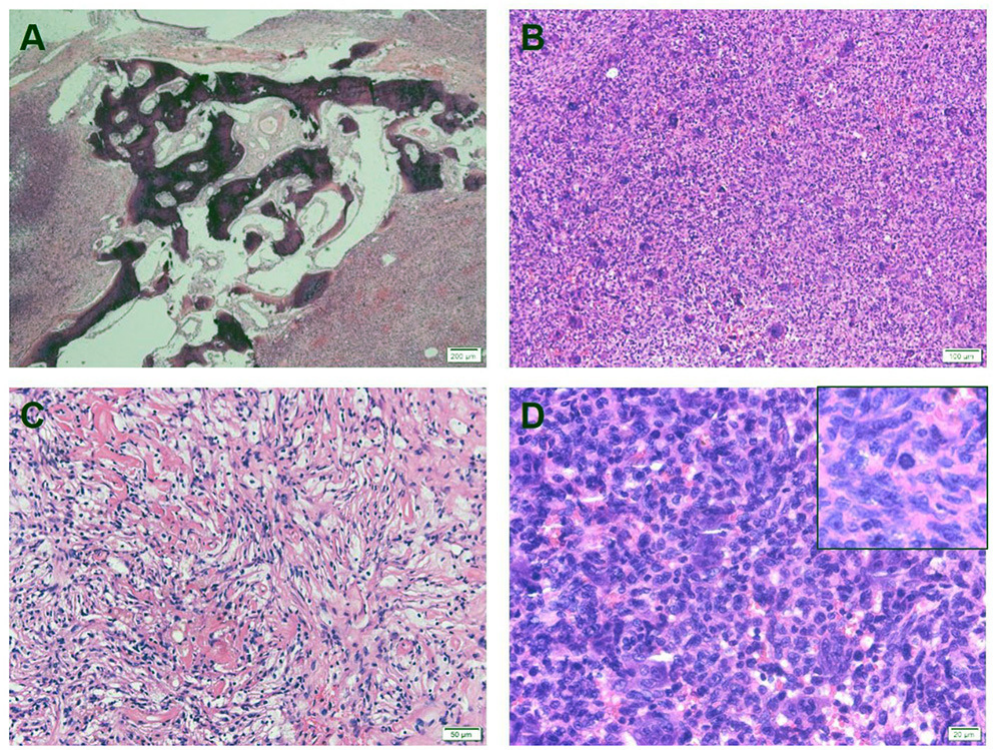
**(A)** Hematoxylin and eosin stain (HE) showed that tumor cells invade bone tissue. **(B)** Tumor tissue consists of mononuclear cells and a large number of evenly distributed osteoblast-like cells. **(C)** A histological pattern of benign fibrous histiocytoma can be seen, with more foam-like cells. **(D)** The nuclear morphology of osteoblast-like multinucleated giant cells is similar to that of mononuclear cells, and nuclear division images are visible (upper right corner).

The histological examination revealed that the lesion was dominated by large numbers of osteoclast-like giant cells between which mononuclear cells were embedded (Figure 2). The giant cells had a variable number of nuclei, some with >50 per cell. Mononuclear cells presented a variety of morphological appearances, including round to oval cells in a nonfibrotic background. Besides, an area of foamy macrophages in a fibrous matrix, which would have previously been called benign fibrous histiocytoma, and focal hemorrhage could be seen (Figure 2).

**Figure 3:**
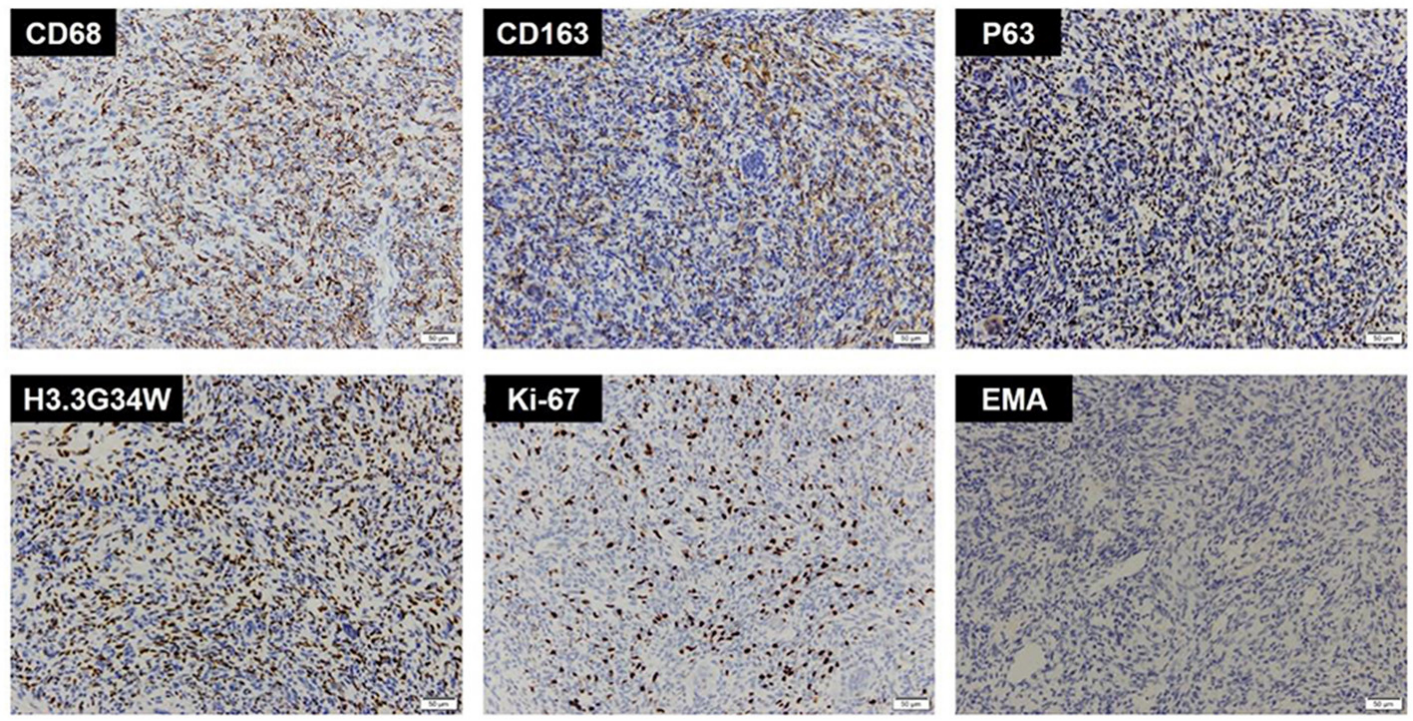
Immunohistochemistry of CD68, CD163, P63, H3.3G34W, Ki-67, and EMA. Immunohistochemistry showed CD68(+), CD163(+), P63(+), H3.3G34W(+), Ki-67(+, 20%), and EMA(-).

An extensive immunohistochemical panel was performed (Table 1). Immunoreactivity for CD68 showed strong cytoplasmic positivity in osteoclast-like giant cells and mononuclear cells. The mononuclear cells showed diffuse P63, CD163, and H3.3G34W immunoreactivity. There was varying immunopositivity for Vim, S-100, and D2-40. Immunostaining was negative for GFAP, EMA, CK, PR, CD34, and E-Cad. The Ki67 proliferation index was around 20–30% (Figure 3). The final pathological diagnosis was GCT of bone in the saddle area.

**Table 1:** Immunohistochemistry results.

**Antibodies**	**Major cell specificity**	**Reactivity**
CD68	Giant cells/ mononuclear cells/ foamy macrophages	+++
CD163	Mononuclear cells/ foamy macrophages	+++
P63	Mononuclear cells	+++
H3.3G34W	Mononuclear cells	+++
Vim	Giant cells/ mononuclear cells/ foamy macrophages	+ (Partial)
D2-40	Giant cells/ mononuclear cells/ foamy macrophages	++ (Focal)
S-100	Mononuclear cells	++ (Scattered)
GFAP	-	-
E-Cad	-	-
PR	-	-
CD34	-	-
CK	-	-
EMA	-	-
Ki67	Mononuclear cells	+++ (Around 20-30%)

*+++ strong reactive; ++ moderate reactive; + weak reactive; - no reactive*.

The patient was admitted to the hospital on June 9, 2021, for a new right thalamic hemorrhage, and physical examination revealed a right-sided actinic nerve palsy, and the patient recovered and was discharged after being given conservative treatment. Unfortunately, the patient died in January 2022 due to the recurrence of a brain hemorrhage.

## Discussion

This report describes a case of a GCT located intracranially, involving the pterygoid sinus, cavernous sinus, and basilar artery, as well as being encircled by the internal carotid artery. The previous imaging findings misled the clinician and led to the diagnosis of the tumor as a meningioma all along. The tumor seen at surgery remained highly similar to a meningioma in general, and so it was not determined to be a GCT of the bone until the final pathology results. In this case, the tumor originated from the clivus, located in the pterygoid sinus and clivus, and destroyed the bones of the clivus so as to invade into the lower part of the right saddle and the cavernous sinus. The size of the tumor in the pterygoid sinus was about 3.2 × 3.6 × 3.0 cm. It was solid, grayish-yellow in color with a clear border, and with a general blood supply and a tough texture.

A giant cell tumor is a rare tumor that is seen in young adults, primarily involves the long bone epiphysis (4, 12), and only rarely occurs in the skull (13, 14). Intracranial GCT is predominantly prevalent in the middle cranial fossa, temporal bone, and skull base (15). GCTs of the skull are mostly located in the middle cranial fossa and originate from the temporal bone or sphenoid bone because the sphenoid–temporal bone undergoes endochondral ossification, while the other cranial bones undergo membranous ossification. GCT may invade the base of the middle cranial fossa, the sphenoid ridge, the cavernous sinus, the saddle area, and even the extensive skull base; outwardly, it may invade the zygomatic arch, the jaws, and the temporalis muscle (16). The GCT in the temporal bone spreads within the lamina cribrosa and shows expansive growth and reactive bony changes. The clinical manifestations of cranial GCT are mainly headache and dizziness, followed by symptoms of cerebral nerve injury, and the invasion of cranial GCT into the skull can cause symptoms of high cranial pressure (16). Cranial GCT located at the base of the skull may invade the trigeminal nerve, abducens nerve, optic nerve, oculogyric nerve, and the facial auditory nerve, resulting in vision loss, eye movement disorders, facial sensory loss, facial palsy, and hearing loss (16).

The cavernous sinus area is a narrow space and has a complex structure. In this area, there are mainly inflammatory diseases, vascular diseases, and neoplastic diseases. Neoplasms include pituitary adenoma, meningioma, schwannoma, lymphoma, perineural tumor spread, metastases, and direct tumor invasion (such as from nasopharyngeal carcinoma) (17). Pituitary adenomas account for ~10% of the cases (18). The characteristic difference between pituitary adenomas and meningiomas is that they do not usually narrow the internal carotid artery (18). Schwannoma appears on imaging as cystic components that may have tubular or linear structures (17). Lymphomas have a high-density shadow on CT. In contrast, nasopharyngeal carcinoma is seen to infiltrate through the skull base adjacent to and invade the cavernous sinus area (19). In the case of metastatic tumors, patients often have imaging evidence of multiple metastases and can find the primary focus of origin. Meningiomas often have characteristic dural attachments and dural tail signs (20), and are among the most common tumors originating in the cavernous sinus region. Meningioma is one of the common intracranial tumors, often occurring in the convex surface of the brain, parsagittal sinus, and pterygoid crest (21). Most meningiomas have characteristic imaging manifestations and are relatively easy to diagnose, but the complex histology of meningioma pathology, differences in biological characteristics, and the possibility of occurrence in rare sites of occurrence have led to diverse imaging manifestations of some meningiomas, which can easily lead to diagnostic difficulties and misdiagnosis (22).

Giant cell tumors of the skull occur most frequently in the sphenoid and temporal bones and very rarely in the frontal, parietal, and occipital bones (16, 23). GCTs of the temporal bone are usually associated with retroauricular pain, conductive hearing loss due to tumor invasion of the infratemporal fossa, and obstruction of the eustachian tube (16). GCTs involving the pterygoid bone are associated with headache, ophthalmoplegia, trigeminal hypesthesia, and visual disturbances (16). GCTs in the saddle area are associated with headache, visual field defects, blindness, diplopia, second to eighth cerebral nerve dysfunction, neck pain, endocrine disorders, and altered mental status (16, 24). There are a few cases in the literature, where GCTs of the bone in the cavernous sinus region are closely related to the meninges and thus misdiagnosed as meningiomas. In this case, the patient had imaging features unique to meningiomas and was in a specific location, leading to misdiagnosis in the final clinical diagnosis. Also, GCT has both benign and malignant tumor characteristics, making it difficult to diagnose solely on the basis of imaging, so it is highly likely to cause misdiagnosis once it occurs in a rare area.

The diagnosis of GCT is done by imaging modalities such as X-rays and MRI (25). MRI is less specific, with tumors showing low to moderate signal intensity on T1 and high signal on T2-weighted images (26). Neither does the whole-body skeletal scintigraphy characterize GCT, nor does the degree of tracer uptake reflect the severity of the tumor. However, whole-body skeletal scintigraphy can help rule out the possibility of multiple GCT metastases (25). In any case, biopsy tissue sampling and histological examination are necessary to support the diagnosis of GCT. In meningiomas, low-grade tumors (WHO type I) have a predominantly slightly low and isosignal T1WI signal. The T2WI signal is often predominantly iso- and slightly high, and relatively high-grade tumors (WHO type II and WHO type III) generally have iso-signal T1WI and T2WI signals (27). In general, the meningeal caudal sign is characteristic of meningiomas to distinguish them from other tumors (28). Therefore, in this case, the presence of the tumor at an atypical site and the presence of the meningeal tail sign could very easily induce the medical staff to diagnose meningioma and also misdiagnose it.

As research progresses, an increasing number of molecular targets are being used as diagnostic targets for GCT. Research evidence suggests that H3.3G34W contributes to GCT by maintaining the transformed state of osteoblast-like progenitor cells, promoting tumor growth, pathological recruitment of giant osteoclasts, and bone destruction (29). Thus, H3.3G34W is a specific indicator for the diagnosis of GCT. The signaling pathway by which osteoblasts induce osteoclast formation was discovered 20 years ago. Receptor activator of nuclear factor kappa B ligand (RANKL) is a tumor necrosis factor family member secreted by osteoblasts, and it binds *via* its receptor (i.e., RANK) to cells of the monocyte lineage to induce osteoclastic differentiation (30, 31). RANKL expression is increased in stromal cells of GCTB and is thought to mediate osteoclast recruitment in developing tumors (32). Mononuclear neoplastic cells can be identified by osteoblast-associated markers such as RUNX2 and P63, positive CD68 and TRAP in mononuclear histiocytic cells, and osteoblast-like multinucleated giant cells are also a basis for identifying different histologies of GCTs of the bone (33). Giant cells are responsible for destructive osteolysis in GCT, including the bone cortex that leads to pathological fractures in about 30% of the patients (34). The metabolic activity of GCT is mainly related to the number of osteoclasts. Giant cells possess bone-resorbing enzymes, including tartrate-resistant acid phosphatase (TRAP), matrix metalloproteinases (MMP2 and MMP9), and cathepsin K (CTSK) (35, 36).

The treatment of localized GCT is primarily surgical, from intralesional curettage with or without local adjuvants to en bloc resection and even amputation (31, 37). Curettage can be performed alone or in combination with local adjuvants that fill the bone cavity. These include bone graft (allogeneic or synthetic composite) and bone cement (polymethylmethacrylate). The current surgical options for intracranial GCT of the bone are mainly based on the intracranial location. Shen et al. reported that 28 patients with GCT of bone invading the lateral skull base in the temporomandibular joint (TMJ) region underwent temporal craniotomy through a lateral temporal craniotomy and a preauricular approach (infratemporal fossa type A) with better outcomes than the traditional intracranial scraping of the skull base lesions recommended (38, 39). For GCT of bone invading the temporal bone alone and invading the temporal bone and pterygoid bone, Feng et al. used a C-shaped preauricular infratemporal fossa approach to achieve a gross total resection and repair of the lateral skull base internal and external communication defect with a pedicled temporal muscle fascial flap (40). Tumors located in the anterior skull base, pterygoid saddle area, orbital apex, and cavernous sinus can be completely removed by an endoscopic transnasal approach (41). Tumors in the infratemporal fossa that do not involve the middle ear, extensive intracranial area, or encircle the temporomandibular joint can also be removed through this approach (42), and even the medial temporomandibular joint can be removed through the nose (43). If the tumor involves extensive middle ear, temporomandibular joint, facial nerve, rocky bone segment, and the following internal carotid artery, the infratemporal fossa approach is required (44), and the anterior and posterior auricular approaches can be used flexibly depending on the extent of tumor involvement anteriorly and posteriorly. In addition to the above-mentioned surgical accesses, several other surgical accesses are mentioned in the literature. These include fisch type II approach (45); temporal craniectomy *via* an extended pterional approach and canal wall down mastoidectomy (46); preauricular approach and parotidectomy (14); extended parotidectomy approach with the sacrifice of the zygomatic arch (47); and modified Obwegeser retromaxillary approach (48, 49). Radiotherapy has been used effectively in cases of GCT with multiple tumor recurrence or difficult surgical treatment (50), and it has been shown to be effective in controlling tumor growth in the vast majority of cases, but with reduced effectiveness in a minority of patients with recurrence. Systemic therapy for GCT includes chemotherapy, interferon, and bisphosphonates (37). Bisphosphonates have a high affinity for the bone matrix and will bind to areas of active bone reconstruction. The uptake of bisphosphonates by bone giant cells activates apoptotic pathways, thereby protecting bone tissue. Denosumab, a fully-human monoclonal antibody used against the high expression of RANKL within GCT, is also currently effective in the vast majority of GCTs (37), but the high cost of treatment still limits access to many. Radiotherapy has been used as a complementary treatment to surgical treatment for patients with inoperable or difficult or unresectable tumors (51). Caudell et al. used a retrospective analysis of 25 patients with pathologically confirmed GCTB who underwent radiotherapy between 1956 and 2000 to suggest that radiotherapy should be considered as an adjunct to surgery or as an alternative therapy in cases of unresectable or resected GCTB that would result in severe functional deficits (52). The current treatment range for patients with GCTs is recommended to be 35–45 Gy (53), as higher than 45 Gy causes an increased incidence of malignancy (54), although in a 2015 study, it was suggested that there was no association between treatment dose and the incidence of malignancy (55). However, a recent study showed that the combination of surgery and chemotherapy did not improve patient survival compared to surgery alone (56). The monoclonal antibody Denosumab has been shown to be sufficiently effective in GCTs, and with radiation therapy causing side effects such as tissue necrosis and central nervous system damage (57), further results are still pending for chemotherapy as a conventional treatment option for patients with GCTs.

## Conclusion

We report a very rare case of intracranial GCT of bone in which the final diagnosis was established by postoperative immunohistochemistry. This report contributes to the scarce literature on these tumors in the skull. The diagnosis of intracranial GCT of the bone is difficult, and we should take into account some more rare tumor types in the diagnosis rather than relying on imaging alone to determine the diagnosis.

## Data Availability Statement

The original contributions presented in the study are included in the article/supplementary material, further inquiries can be directed to the corresponding authors.

## Ethics Statement

Written informed consent was obtained from the individual(s) for the publication of any potentially identifiable images or data included in this article.

## Author Contributions

JY and SH wrote the article. SH and XL collected and edited the pathology imaging. JY, SC, and YWu collected clinical case information and imaging data. JY, SC, and YWu mainly work on collecting clinical case information and imaging data. All authors approved the final study.

## Funding

This study was supported by the Natural Science Foundation of Hainan Province (820QN387), the National Natural Science Foundation of China (NSFC No. 81860347), the Hainan Province Science and Technology Special Fund (ZDYF2021SHFZ238), the Innovative Research Projects for Postgraduates in Higher Education Institutions in Hainan Province (Hys2020-342), the Hainan General Hospital Natural Science Foundation Incubation 530 Project (2021MSXM13), and Hainan General Hospital Hospital-level Youth Fund Project (QN202016).

## Conflict of Interest

The authors declare that the research was conducted in the absence of any commercial or financial relationships that could be construed as a potential conflict of interest.

## Publisher's Note

All claims expressed in this article are solely those of the authors and do not necessarily represent those of their affiliated organizations, or those of the publisher, the editors and the reviewers. Any product that may be evaluated in this article, or claim that may be made by its manufacturer, is not guaranteed or endorsed by the publisher.
